# Number of prior negative screening outcomes does not influence future risk of breast cancer

**DOI:** 10.1007/s10654-020-00645-0

**Published:** 2020-05-19

**Authors:** Marie Lilleborge, Ragnhild S. Falk, Solveig Hofvind

**Affiliations:** 1 Section for Breast Cancer Screening, Cancer Registry of Norway, Postbox 5313, Majorstuen, 0304 Oslo, Norway; 2 Oslo Centre for Biostatistics and Epidemiology, Oslo University Hospital, Oslo, Norway; 3 Department of Life Sciences and Health, Oslo Metropolitan University, Oslo, Norway

**Keywords:** Breast cancer, Mammography, Risk assessment, Screening history

## Abstract

We questioned whether a history of negative screening outcomes could be used to predict breast cancer risk, and thus be used as a potential factor for stratification of mammographic screening. Data from the Norwegian population based breast cancer screening program, BreastScreen Norway, was used to estimate cumulative hazard rates for breast cancer by number of prior negative screening outcomes among participants from 1995 through 2016. We followed three age cohorts of women, who started screening at age 50–54, 55–59, and 60–64 years. Further, we estimated the absolute and relative risk of breast cancer by number of prior negative screening outcomes. The cumulative hazard curves were parallel for all numbers of negative screening outcomes for all age cohorts. The absolute risk of breast cancer increased with number of negative screening outcomes for the youngest age cohort. For the oldest age cohorts, the absolute risk was stable during the screening period and decreased thereafter. The number of negative screening outcomes was not associated with risk of breast cancer, adjusted for age, percent screening attendance and calendar years (HR 1.00, 95% CI 0.98–1.02). Our results suggest that the number of negative screening outcomes does not predict breast cancer risk among participants in BreastScreen Norway.

## Introduction

Mammographic screening is aimed at reducing breast cancer mortality by detecting tumors at an early stage. A reduced rate of breast cancer is expected for a time after a negative screening outcome because of earlier diagnoses due to screening. Comparison of screened and unscreened populations in a modelling study has in fact shown that excess age- and time-specific cumulative incidence was expected to nullify after 30 years of follow-up [1].

Stratification is a hot topic in cancer screening research, and refers to reduced testing in lower-risk groups as well as intensified testing in higher-risk groups. Stratified mammography screening based on risk factors is suggested to make the screening more effective both for the women and for the society [2–4].

Inspired by Walter and Day [5], Andersen et al. introduced a hypothesis that women with a certain number of prior negative screening outcomes could be identified as a low risk group [6]. They used data from organized mammographic screening in Sweden and Denmark, and found negative screening outcomes were not a predictor of breast cancer risk, and therefore not suitable for stratified screening. For the purpose of complementing, replicating, and potentially expanding the generalizability of the findings by Andersen et al., we wanted to investigate the same research question. Further, we wanted to explore this by other methods, adding additional novelty to this study.

## Materials and methods

Information about attendance and screening outcome in BreastScreen Norway was extracted from the Cancer Registry of Norway, which is administrative responsible for the population based screening program [7]. The Cancer Registry databases include among others information on date of invitation, attendance, screening outcome, breast cancer diagnosis, and vital status. By law, all cancer cases in Norway are reported to the Cancer Registry, ensuring complete capture of cancer [8]. This allows us to follow each screened woman for breast cancer regardless of her adherence to the screening program and potential moves between counties.

BreastScreen Norway started in 1995 and became nationwide in 2005 [7]. The program serves approximately 650,000 women who are offered biennial independent double-read, two-view mammographic screening. All women born in birth cohorts corresponding to age 50–69 years at the start of regional screening rounds receive a personal invitation letter with a scheduled time and place for a screening examination. Three out of four invited women attended each screening round, and 84% of all invited women had attended at least once [7]. In our study, we included women aged 50–64 years at first attendance in BreastScreen Norway during the study period (1995–2016).

The Cancer Registry Regulations waive the requirement to obtain written informed consent for this retrospective analysis of de-identified data from BreastScreen Norway. No institutional review board approval was required.

### Definitions

A *breast cancer* was considered as carcinoma in situ (lobular carcinoma in situ, LCIS; ductal carcinoma in situ, DCIS) or invasive breast cancer (ICD-10 C50).

A screening examination was considered negative if the women were not recalled for further assessment, while a *negative screening outcome* was defined as a screening examination not resulting in a diagnosis of breast cancer independent of recall. That is, a negative screening outcome could include a false-positive screening examination resolved at recall by additional imaging only, or additional imaging including biopsy.

Detection mode was defined as *screen*-*detected* (diagnosed within 6 months after a positive screening examination), *interval detected* (diagnosed within 24 months after a negative screening examination or 6–24 months after a positive screening examination) or *outside the screening program* (diagnosed more than 24 months after the prior screening examination).

The *screening interval* in BreastScreen Norway is two years. In the study period, the median time between two screening invitations was 731 days (interquartile range 720–746 days) [7]. A *regular screening pattern* was defined as a series of screening examinations in which each pair of consecutive screens was spaced with 1.5–2.5 years in between.

We defined three cohorts depending on age at first screen in BreastScreen Norway: *Age cohort 1* consisted of women aged 50–54 years at date of first screen, *age cohort 2* consisted of women aged 55–59 years at date of first screen, and *age cohort 3* consisted of women aged 60–64 years at date of first screen.

### Covariates

*Age at screening* was calculated from the date of screening attendance and the woman’s date of birth (available for all women in the study). *Percent screening attendance* for a woman after her *N-*th attended screen was estimated as *N* divided by the total number of invitations received. If two consecutive screens were at least four years apart, we estimated that the woman had declined one invitation in the time interval, and similarly two invitations if six years apart, and so on.

### Study population

We received data about 762,425 women with no prior diagnosis of breast cancer before first attendance in BreastScreen Norway and whose first screening outcome was negative with at least 6 months of follow-up. We excluded 62,542 women who were 48 or 49 years at first screen and 70,620 women who were 65 years or older at first screen. This resulted in a study population of 425,804 women in age cohort 1 (aged 50–54 years at first screen), 118,956 women in age cohort 2 (aged 55–59 years at first screen) and 84,503 women in age cohort 3 (aged 60–64 years at first screen).

## Statistical analysis

We used three different approaches to evaluate whether women with a certain number of prior negative screening outcomes could be identified as a low-risk group. We used a cumulative hazard rate of breast cancer as described in Andersen et al. [6]. Additionally, we estimated the absolute and relative risk of breast cancer.

For all three approaches, we followed the women longitudinally from first attendance in BreastScreen Norway until breast cancer, independently of detection mode. Women were censored at the end of follow-up (December 2016), at the age of 80 years (a maximum of 10 years after she aged out of the screening program), or at the diagnosis of another type of cancer located in the breast.

To estimate the cumulative hazard rate of breast cancer by age cohort, each woman’s follow-up time was analysed by the number of prior negative screening outcomes. We classified follow-up time to number of negative screening outcome groups as follows: One negative screening outcome: from first negative screening outcome until second negative outcome, date of breast cancer diagnosis, death, emigration or end of follow-up, whichever date came first. Two negative screening outcomes: from second negative screening outcome until third negative outcome, date of breast cancer diagnosis, death, emigration or end of follow-up, whichever date came first. And so on. These definitions ensured that a women with *N* negative screening outcomes, who had a screen-detected breast cancer at her next screen, remained in the *N* negative screening outcome group, and her screen-detected breast cancer counted as an event for the *N* negative screening outcome group. As a woman contributed follow-up time to a unique group at each time point and follow-up time was censored after diagnosis of breast cancer, each breast cancer case was assigned to a unique negative screening outcome group.

For each age cohort, the cumulative hazard rate of breast cancer was calculated as a function of time since first screen, and by number of negative screening outcomes. As in Andersen et al. [6], we used visual inspection to evaluate whether the cumulative hazards from two groups were proportional from a certain point in time, indicating that the two groups had the same breast cancer risk from that point on.

A considerable proportion of breast cancers were screen detected, thus the cumulative hazard of the *N* negative screening outcomes group increased around the time when the women were screened again (e.g. around 2 years after the start of the curve). As in Andersen et al. [6], we focused on the comparison between groups of women with *N − 1* negative screening outcomes and *N* negative outcomes in the time period more than 2 years after ‘the start of the curve’, to exclude possible screen-detected breast cancers in the following screening round. Sudden jumps in the cumulative hazard curve were typically related to mathematical instabilities as women gradually entered or a large fraction left the group. These sudden jumps in a curve should be ignored as we focus on proportionality of curves from after the time of the next screening round.

The absolute risks of breast cancer by number of negative screening outcomes was presented as incidence rates (IRs) per 1000 woman-years with 95% confidence intervals (CIs). In these analyses, the women were followed from *N*th negative screening outcome until breast cancer or end of follow-up regardless of subsequent screening attendance. This is in contrast to the cumulative hazard analyses, as a woman could contribute follow-up time to several negative screening outcome groups simultaneously, e.g. a woman diagnosed with screen-detected breast cancer at the 4th screening examination had her outcome assigned to three negative screening outcome groups. This approach resulted in a lower follow-up time after the third screening outcome compared to the first. The motivation for this approach is related to the contrast between the inherent assumption of independent censoring in survival analysis, and the underlying hypothesis of this paper; that women with several negative screening outcomes are at lower risk of breast cancer.

The relative risk of breast cancer was presented as hazard ratios (HRs) by number of negative screening outcomes. The HRs were estimated by Cox regression with number of negative screening outcomes (exposure) as a time-varying covariate, adjusted for percent screening attendance, calendar year (time-varying covariates) and age.

Data preparations, analyses and visualizations were performed using Stata (version 16, StataCorp, College Station, TX, USA).

## Results

We followed 629,263 women who had 2,794,013 negative screening outcomes from 1995 through 2016. In age cohort 1, we identified 11,886 breast cancer cases among 425,804 women during a median follow-up of 9.9 years. In age cohort 2, there were 5252 breast cancer cases among 118,956 women during a median follow-up of 14.3 years, and in age cohort 3 there were 3282 cases among 84,503 women during a median follow-up of 14.7 years. In age cohort 1, 30% of the women had their first screen in calendar year 1995–2002, 35% in 2003–2009 and 35% in 2010–2016. In age cohorts 2 and 3, more than 85% of the women had their first screen during the staggered implementation of BreastScreen Norway, 1995–2005. Among women diagnosed with breast cancer during follow-up, the median time to breast cancer was 2.2 years from the previous negative screening outcome (interquartile range 2.0–2.5 years), and 7.5 years from the first negative screening outcome (interquartile range 4.1–11.1 years).

The number of women contributing follow-up time within each age cohort decreased with the number of negative screening outcomes (Table 1). This followed by definition, as a woman contributing follow up-time to the *N* negative screening outcomes group must necessarily have contributed follow up-time to the *N − 1* negative screening outcomes group prior to her current screen. The proportion of breast cancers outside the screening program first declined with number of screening outcomes (within *N ≤ 7* in age cohort 1, *N ≤ 5* in age cohort 2 and *N ≤ 2* in age cohort 3). Thereafter, the proportion increased by *N*, similar to the increased proportion of the women who gradually aged out of the program.

**Table 1:** Number of women in age cohorts by number of prior negative screening outcomes, number of screen-detected breast cancer, number of symptomatic breast cancer and total number of breast cancer for BreastScreen Norway, 1995–2016

Negative screening outcome	Women	Total number of breast cancers	Screen-detected breast cancer		Interval detected breast cancer		Outside the screening program	
(n)	(n)	(n)^a^	(n)^b^	%	(n)^c^	%	(n)^d^	%
*Age cohort 1 (50*–*54* *years at 1st screen)*								
1	425,804	2560	1423	56	747	29	390	15
2	352,715	2143	1363	64	580	27	200	9
3	295,600	1887	1270	67	475	25	142	8
4	244,716	1589	1112	70	379	24	98	6
5	197,506	1349	961	71	330	25	58	4
6	153,220	1114	817	73	259	23	38	4
7	109,726	649	460	71	161	25	28	4
8	66,320	380	232	61	90	24	58	15
9	30,647	215	98	45	68	32	49	23
*Age cohort 2 (55*–*59* *years at 1st screen)*								
1	118,956	997	591	59	231	23	175	18
2	107,266	863	587	68	175	20	101	12
3	99,169	842	553	66	202	24	87	10
4	91,473	819	557	68	162	20	100	12
5	82,576	802	490	61	169	21	143	18
6	67,101	622	241	39	126	20	255	41
7	34,356	247	51	21	52	21	144	58
8	6673	60	2	3	13	22	45	75
*Age cohort 3 (60*–*64* *years at 1st screen)*								
1	84,503	748	443	59	145	19	160	22
2	76,210	693	448	65	118	17	127	18
3	68,145	849	346	41	114	13	389	46
4	48,061	627	128	21	83	13	416	66
5	21,518	332	16	5	48	14	268	81
6	2349	33	0	0	5	15	28	85

^a^Total number of breast cancers: sum of screen detected, interval and breast cancers outside the screening program

^b^Screen-detected breast cancers: detected < 6 months after a positive screen

^c^Interval breast cancers: detected < 24 months after a negative screen or 6–24 months after a positive screen

^d^Breast cancers outside the screening program: detected more than 24 months after the prior screen

**Fig. 1:**
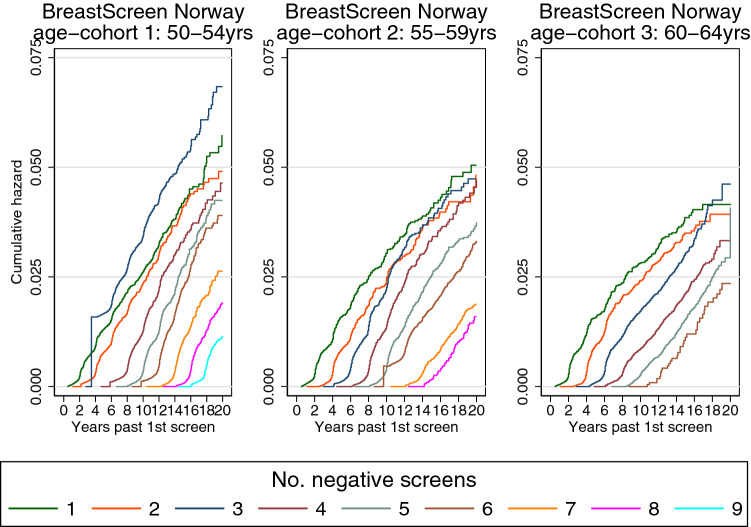
Cumulative hazard of breast cancer by time since first screen and number of negative screening outcomes among participants of BreastScreen Norway, 1995–2016. Women aged 50–54 (55–59) 60–64 years at 1st screen were included in age cohort 1 (2) 3

For all age cohorts, the cumulative hazards from two groups (*N* and *N*-*1* negative screening outcomes) were parallel from *N* times 2 years after first screen (Fig. 1). That is, the future risk of breast cancer did not depend on the women’s number of prior negative screening outcomes.

The absolute risk of breast cancer increased by number of negative screening outcomes, from 3.0 to 3.5 per 1000 woman-years, for age cohort 1 (Table 2). For age cohorts 2 and 3, the absolute risk was stable during the screening period (N ≤ 5 in age cohort 2 and N ≤ 2 in age cohort 3), while it decreased thereafter for age cohort 3.

**Table 2:** Crude Incidence Rates per 1000 woman-years (IR) with 95% Confidence Interval (CI) from Nth negative screening outcome until breast cancer or end of follow-up, 1995–2016

N^a^	Age cohort 1 50–54 years at 1st screen	Age cohort 2 55–59 years at 1st screen	Age cohort 3 60–64 years at 1st screen
	IR (95% Cl)	IR (95% Cl)	IR (95% Cl)
1	3.0 (2.9–3.0)	3.3 (3.2–3.4)	2.9 (2.8–3.0)
2	3.1 (3.1–3.2)	3.3 (3.2–3.4)	2.8 (2.7–2.9)
3	3.3 (3.2–3.3)	3.3 (3.2–3.5)	2.7 (2.5–2.8)
4	3.4 (3.3–3.5)	3.3 (3.2–3.4)	2.4 (2.3–2.6)
5	3.5 (3.4–3.6)	3.1 (3.0–3.2)	2.4 (2.2–2.7)
6	3.5 (3.3–3.6)	2.7 (2.5–2.9)	2.5 (1.7–3.4)

^a^No. negative screening outcomes prior to inclusion

The number of negative screening outcomes was not associated with breast cancer when adjusted for age, percent attendance and calendar year (HR 1.00, 95% CI 0.98–1.02, Table 3).

**Table 3:** Hazard ratio (HR) of breast cancer with 95% Confidence Interval (CI) by number of prior negative screening outcomes (time-varying covariate), 1995–2016

	Univariable^a^ HR (95% CI)	Multivariable^a^ HR (95% CI)
No. prior negative screening outcomes	0.99 (0.98–0.99)	1.00 (0.98–1.02)
Screening attendance [per 10% increase]	1.00 (1.00–1.00)	1.00 (1.00–1.00)

^a^Adjusted (linear) for age at first screen and calendar year at screening attendance

### Sensitivity analyses

**Fig. 2:**
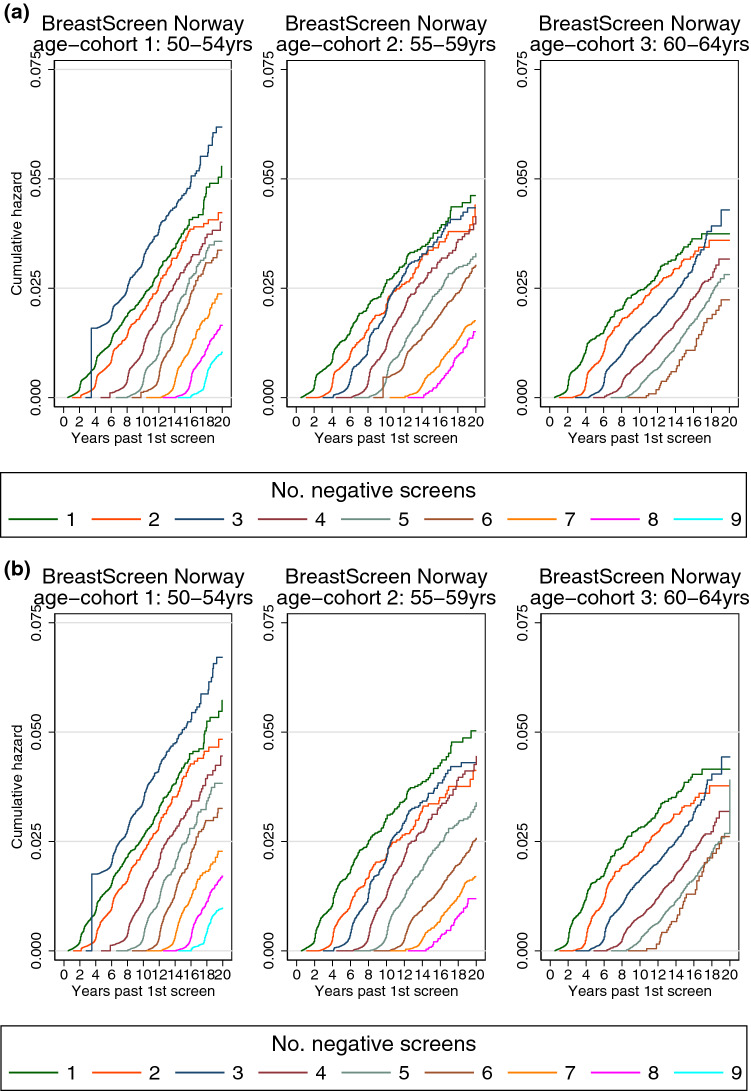

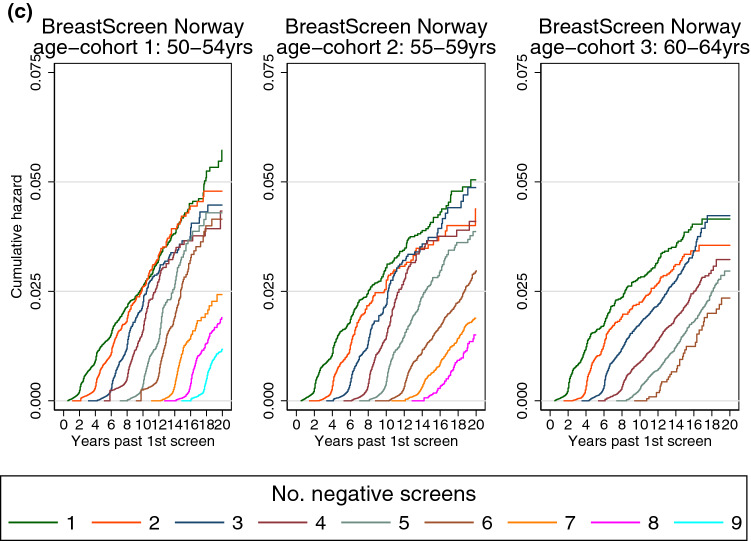
Sensitivity analyses of cumulative hazard of breast cancer by time since first screen and number of negative screening outcomes among participants of BreastScreen Norway, 1995–2016 (main analysis in Fig. 1). Panel a: Women are followed for invasive breast cancer, and censored at diagnosis of carcinoma in situ due to treatment. Panel b: Women are censored after screening examinations including a recall. That is, the number of negative screening outcomes are counting only negative screens without a recall. Panel c: Women are censored just before the next screen if it is < 1.5 year or > 2.5 years after the previous screen. That is, we are only following women with a regular attendance pattern

Three sensitivity analyses were performed for the cumulative hazard analyses. First, when the women were followed for invasive breast cancer (excluding carcinoma in situ as outcome), the number of outcomes decreased from 20,420 breast cancers to 17,936 invasive breast cancers. The cumulative hazards of breast cancer remained parallel from *N* times 2 years after first screen (Fig. 2a). Second, the cumulative hazard of breast cancer appeared unchanged when the exposure was the number of negative screening examinations without recall (Fig. 2b). Third, when we restricted the analysis to women with a regular attendance pattern, some sudden jumps at the start of the curve are removed (Fig. 2c).

## Discussion

We did not observe any reduction in breast cancer risk among women with high versus low number of negative screening outcomes, in BreastScreen Norway. We used the same study design as Andersen et al. [6], and had similar access to complete, individual-level data on screening history and follow-up. Our study confirmed the conclusions in their study, with remarkably similar results. Further, additional analyses strengthened our findings.

We followed three age cohorts, 50–54, 55–59 and 60–64 years at first screen in BreastScreen Norway, to diagnosis of breast cancer or end of follow-up. The decline in proportion of breast cancers detected outside the screening program by number of negative screening outcomes could be related to an increased adherence to the program by number of accepted invitations. Since 2005, when the program was fully implemented, age of first invitation to screen is roughly 50 years for all women. Thus, the median follow-up time was lower for women in age cohort 1 compared to age cohorts 2 and 3.

The crude absolute risk of breast cancer increased by number of prior negative screening outcomes in age cohort 1. The findings could be related to limited follow-up time and the increased risk of breast cancer by age. The stable risk of breast cancer during the screening period for age cohorts 2 and 3 might be related to the fact that women were contributing the same follow up time in several number of negative screening outcome groups. The decrease observed in age cohort 3 might indicate that the women gradually aged out of the program.

One weakness of the study was that we, in contrast to Andersen et al. [6], did not study a fixed cohort. Our study included all women whose first screening examination in BreastScreen Norway was negative. This resulted in a larger study population compared to Andersen et al. [6], whose analysis was limited to women aged 50–64 years at start of the program. Thus, a strength of our study was the large study sample, consisting of 629,263 women followed for up to 21 years.

We did not have available information about lifestyle and socioeconomic variables. However, in our Cox regression, we did take into account proportion of invitations where the women did show up for screening (percent attendance) and also calendar year at screen to account for increased sensitivity of the program and the screening equipment over time. A strength of our study was that in addition to the visual inspection inspired by Andersen et al. [6], we presented incidence rates by number of negative screening outcomes and a Cox regression model with number of negative screening outcomes as a time-varying covariate to support our interpretation of the cumulative hazard curves.

In conclusion, we found the number of negative screening outcomes not to be associated with the risk of breast cancer. Our results indicate that history of negative screening outcomes cannot be used as a predictor for breast cancer risk among participants in BreastScreen Norway.
